# Remarkable Case of Corpuscles Freely Moving Both in the Vitreous Humor and in the Posterior Chamber of the Eye, and Causing the Imaginary Perception of Objects

**Published:** 1829-02

**Authors:** 


### CORPUSCLES IN THE EYE.

*Remarkable Case of Corpuscles freely moving both in the Vitreous Humor and in the posterior Chamber of the Eye, and causing the imaginary Perception of Objects.*

On the 17th August, M. Galy, surgeon of the Hospital of Périgneux, consulted M. Parfait-Landrau on the case of M. Audebert, formerly a magistrate, then in his seventieth year, and of a bilio-sanguineous temperament: he was subject to wandering pains, resembling those of rheumatism, and had for several years experienced an alteration in regard to the sight of his right eye, of which he feared the consequences, although the affection had remained a long while stationary. He sees muscæ volitantes, black points, and other images, of various shapes, &c., which have been so well described by M. Demours, in a memoir which he recently read to the Academy.

After an attentive examination of the patient's pupils, which were rather contracted, M. Parfait-Landrau thought that he could perceive certain corpuscles moving apparently at the bottom of the posterior chamber of the right eye, and shining with a sort of phosphoric brilliancy. As these phenomena were new, and of a most interesting description, he did not hastily admit the evidence of his senses, but doubted the correctness of first thoughts; and, on the supposition that what appeared to be in the eye might be really nothing more than the reflection of external objects, (although they were not apparent in the sound eye,) he proposed to the patient and his medical attendant that the pupil should be dilated by the extract of belladonna. The pupil was by this means thoroughly dilated, and MM. Parfait-Landrau and Galy distinctly perceived a considerable number of corpuscles, which in general resembled finely powdered liquorice, and a few had the brilliancy of fine gold dust. These particles moved to and fro throughout the whole extent of the posterior chamber, when the eye became fixed, they descended; when it moved, they were again agitated as before; and thus on in succession.

M. Parfait-Landrau is firmly of opinion that these substances were in the vitreous humor, as they were numerous and sufficiently near to be distinctly seen with the naked eye, although he employed a magnifying glass in his examination of them.

This new discovery of an evident and physical cause for that which the ancients called *perpetual imagination*, which the moderns have since attributed to the state of the internal membranes of the eye, to varicose veins in its humors or membranes, is the more remarkable, as no similar phenomenon is described in any work professing to treat of these matters. M. Demours thinks that one of the causes of these muscæ volitantes is due to the humor of Morgagni, in which he supposes there are small portions which, without losing their transparency, become more dense, ponderous, and refractive. Other practitioners, equally respectable, consider them to be produced by the aqueous humor; and our author allows that they cannot, in every case, result from the phenomenon which is the subject of the present paper. He therefore does not attempt to refute the various opinions to which they have given rise. He agrees with M. Demours in stating that these corpuscula volitantia rise with the movement of the eye, but immediately afterwards fall to its most dependent part, whatever may be the precise position of the eye itself. This curious fact may be readily explained in the present patient, but certainly it is not quite so intelligible in those cases where it is attributed to the development of varicose veins in the humors or internal membranes of the eye. It is alike inexplicable on the supposition that it is the effect of partial paralysis of the retina.

In order that these corpuscles might move about in the vitreous humor, the hyaloid membrane which forms its cells must be first destroyed; the natural consequence of which is a considerable reduction in the density of that humor; and it is well known that this alteration may exist without preventing the other parts of the eye from performing their respective functions. For there is scarcely an oculist in the habit of operating for cataract by extraction of the lens, who has not found in some patients the vitreous almost as fluid as the aqueous humor, without preventing the success of such operations. And thus we see that the crystalline humor may, without evident cause, entirely dissolve within its membranous sac, and occasion no alteration in other parts of the eye.

That, in the case of M. Audebert, these corpuscles which appear to move in the vitreous humor, are not contained in its cells, and do not owe their movement to the undulation of this humor, (if it should be deemed capable of undulation,) is proved by the fact that, when the eye moves, they are seen very distinctly rising from the bottom, and traversing the whole posterior chamber. Again, as soon as the eye is stationary, they are seen descending to their former situation; upon which the eye becomes clear, and the patient no longer perceives the muscæ volitantes.

This is certainly a singular phenomenon; but it might, perhaps, have been occasionally observed if the pupils had been always artificially dilated in the examination of similar cases.

M. Audebert reads with the affected eye, and feels no pain in it: the pupil duly contracts and dilates, and all the parts of his eye, the vitreous humor excepted, present no morbid appearances.

Four days after the first investigation, the eye was again examined in the presence of MM. Galey and Renaud, surgeons, and of Dr. Vidal, member of the medical jury of the department, and head physician to the hospital; all of whom testify to the truth of the above statement.

